# Otago exercise programme—from evidence to practice: a qualitative study of physiotherapists’ perceptions of the importance of organisational factors of leadership, context and culture for knowledge translation in Norway

**DOI:** 10.1186/s12913-020-05853-8

**Published:** 2020-10-27

**Authors:** Hilde Worum, Daniela Lillekroken, Birgitte Ahlsen, Kirsti Skavberg Roaldsen, Astrid Bergland

**Affiliations:** 1 Department of Physiotherapy, Faculty of Health Sciences, Oslo Metropolitan University, Postbox 4, St. Olavs Plass, 0130 Oslo, Norway; 2 Department of Nursing and Health Promotion, Faculty of Health Sciences, Oslo Metropolitan University, Oslo, Norway; 3 Department of Neurobiology, Health Sciences and Society, Karolinska Institute, Stockholm, Sweden; 4 Department of Research, Sunnaas Rehabilitation Hospital, Oslo, Norway

**Keywords:** Culture, Evidence-based practice, Fall prevention, Leadership, Norway, Organisational factors, Professional roles, Qualitative research, Thematic analysis

## Abstract

**Background:**

Falls and fall-related injuries are a major public health problem and an international priority for health services. Despite research showing that evidence-based fall prevention is effective, its translation into practice has been delayed and limited. Although organisational factors such as leadership, culture and context are key factors for implementing evidence-based practice, there is still limited information on whether these factors represent barriers in the Norwegian and international healthcare context. Thus, this study aimed to explore the views of physiotherapists in clinical practice and their leaders’ views on the importance of organisational factors, such as leadership, culture and contextual and human resources, regarding successful knowledge translation of the Otago evidence-based fall programme in a Norwegian community.

**Methods:**

Four in-depth interviews with physiotherapists and a focus group interview with nine physiotherapists and leaders representing local hospitals and municipalities were conducted to collect data. The data were analysed using a thematic analysis.

**Results:**

The analysis yielded an overarching theme: an empowering leader as an anchor is needed for successful knowledge translation of physiotherapists’ and leaders’ views about the role of organisational structure, leadership, culture, financial resources and competence in research-based knowledge, as well as how to enhance the clinical staff’s expertise. Four main themes further elaborated on the overarching theme: (1) multifactor leadership—the importance of reinforcement, knowledge, goals and attention; (2) potential for change in professional roles as shaped by culture, context and type of practice; (3) knowledge translation—the tension between real-life capabilities, optimism and learning; and (4) different types of support—environmental resources and social influences.

**Conclusions:**

This study highlighted the importance of organisational factors in knowledge translation in fall prevention. The findings emphasise the importance of leaders’ role and style in providing a supportive culture and contextual factors during the knowledge translation process. This study provides an understanding of the knowledge translation and sustainability of evidence-based practice and the Otago exercise programme for fall prevention programmes for community-dwelling older adults in Norway.

**Supplementary Information:**

**Supplementary information** accompanies this paper at 10.1186/s12913-020-05853-8.

## Background

Worldwide, the pace of the ageing population is increasing dramatically and presents health, long-term care and welfare systems alike with new challenges. Today, 125 million people are aged 80 years or older, and by 2050, the world’s population aged 60 years and older is expected to total 2 billion [1]. The same trend is seen in Norway. In 2017, the number of older adults above 67 will increase from 0.79 million to 1.28 million in 2040 [2] and approximately one-third of community dwellers older than 65 years will experience a fall annually [3, 4]. Falls and fall-related injuries have consequences, both for the individual and for society. Therefore, fall prevention among older adults has emerged as an international health priority in health services [5–7].

Evidence-based multifactorial interventions comprising, for example, exercise, home safety adjustments and medication evaluations can be beneficial in reducing the number of falls and the risk of falling compared with the usual care or attention control [8–10]. However, exercise as a single intervention seems to be the most effective in preventing falls in older adults, which is true also from a cost-efficiency and public health perspective [11, 12].

The Otago Exercise Programme (OEP) is an evidence-based training programme designed specifically to prevent falls in older, community-dwelling adults [13]. The training programme is individualised and consists of exercises that promote strength, balance and walking. During the intervention period, patients receive up to five home visits, as well as monthly phone calls.

Although research has shown that evidence-based fall prevention is effective [3, 13, 14], the translation into practice has been delayed and limited [5, 15–17]. Several researchers have underscored the need to bridge the gap between evidence-based knowledge and practice in the field of fall prevention to enhance the knowledge translation of evidence-based fall prevention programmes [15, 18–21]. Given the high-level evidence supporting the OEP for people who have experienced falls in the past [13], it is timely to develop effective processes to move this research-based programme into practice, which, to the best of our knowledge, has not been addressed before. Organisational factors such as leadership, culture and contextual and human resources might be important for successful knowledge translation (KT). Evidence-based practice (EBP), research utilisation (RU) and KT are familial concepts that refer to the identification, utilisation and application of knowledge from research sources to clinical practice. EBP has been defined as ‘the integration of clinical expertise, patient values, and the best research evidence into the decision-making process for patient care’ [22]. According to Bussieres et al. [23], RU is a part of EBP and refers to the ‘process by which specific research-based knowledge is implemented in practice’. KT, on the other hand, emphasises the synthesis, dissemination, exchange and application of knowledge from research findings and other sources as a way to influence changes in practice and improve health outcomes [24]. Grimshaw et al. [25] have defined KT as ‘ensuring that stakeholders are aware of and use research evidence to inform their health and healthcare decision making’. Thus, KT aims to bridge the gap between research findings and what is routinely done in practice. KT might embrace an approach to RU that recognises that knowledge is created by active and engaged users, often in a nonlinear and emergent fashion [26]. As stated above, the research–practice gap regarding KT and evidence-based exercise intervention such as OEP in fall prevention is a challenge for clinical practice. This may be related to the clinical trials conducted with limited target groups and in ideal contexts, such as highly selective patients, additional resources and highly educated specialised researchers. The effectiveness of such interventions in the primary health care service is an understudied research area within health research, and few—mostly small—studies have included older adults. However, such studies are necessary to ensure that the procedures still have the desired effects when delivered in today’s healthcare system.

It has been increasingly recognised that supportive leadership behaviours are necessary for the organisational institutionalisation of EBP [27, 28]. The contextual factors may interact, influence, modify, simplify or limit the intervention and its effectiveness or equally contribute to the success of the intervention [29]. Despite this, Squires et al. [29] claim that context is often a neglected problem in implementation science. Here, organisational support can facilitate EBP [26, 30]. Gifford et al. [31] systematically reviewed qualitative and quantitative evidence concerning the relationships between managers’ leadership and allied health personnel’s behaviours use of research in clinical practice. Their findings conclude that manager–staff dyads are influential. Furthermore, leadership involves change and task-oriented behaviours that influence the environmental milieu and the organisational infrastructure that supports clinical care. The most frequently cited and impactful organisational barriers include a lack of administrative and cultural support (e.g., resistance from colleagues, leaders and other professions) and insufficient resources [32, 33].

Currently, as stated in a recent publication, there is limited information about the perceived barriers that may hinder the translation of the evidence-based fall prevention programme OEP in the Norwegian and international healthcare context [34]. The feedback provided by the patients and physiotherapists suggests that the OEP can guide clinical practice. Specifically, the participants (i.e., both patients and physiotherapists) noted that the evidence-based knowledge that the OEP entails is relevant, powerful, effective and safe for use when it is adapted to meet the unique needs of the patient and when it is communicated using user-friendly language [34]. A key feature regarding KT is that it brings into focus the organisational, structural, financial and professional strategies when translating evidence into practice [35, 36]. Understanding healthcare providers’ experiences using evidence from their own settings will facilitate better translation of research into practice [37]. Thus, the current study aimed to explore the views of physiotherapists in clinical practice and their leaders’ views on the importance of organisational factors, such as leadership, culture and contextual and human resources, regarding the successful KT of the Otago evidence-based fall prevention programme into the community. The results of the current study can be used to promote KT in fall prevention healthcare services. It is hoped that the findings may facilitate the delineation of improved strategies that can inform policymakers, educators, clinicians, future researchers and older adults.

## Methods

### Design

The present study is inspired by a phenomenological approach, which it uses as a philosophical framework to explore and describe physiotherapists’ views and lived experiences. Phenomenology focuses on how individuals perceive the world [38] and take an interest in psychological processes: respondents’ perceptions, awareness and consciousness [39], all of which were especially pertinent to the research question. Health, health care and health services delivery are subjective phenomena that are understood, enacted and experienced by human beings. An increased understanding of human complexity and multidimensionality (consciousness, subjectivity, intentionality and actions) is crucial if the health system is to optimise the quality of health care and delivery of health services [40]. We aimed to understand the complexity of the factors that influence physiotherapists’ experiences of and reflections on how organisational factors such as leadership, culture and contextual and human resources are of importance for the successful use of research-based knowledge in fall prevention in a community through their perspective and experiences.

### Recruitment and participants

The last author recruited the physiotherapists from one hospital in Norway, while the physiotherapist who worked in the geriatric ward recruited the community physiotherapists who administered the OEP. All physiotherapists were provided with verbal and written information about the study’s aims and the associated data collection methods. Inclusion was based on the following criteria: employed at a geriatric department in a hospital or a primary health service in the community where the patients lived and where the facility was providing OEP within the specified time frame of the study (1 year). We aimed to recruit all the physiotherapists involved in performing and completing the OEP. At the time of study initiation, only four patients had completed the intervention, and their physiotherapists, who administered the OEP, were included.

A purposive sample comprising 12 physiotherapists was chosen. All the participants were ethnic Norwegians. The number of participants was guided by Malterud’s model of information power, suggesting saturation as being a process [41]. According to Malterud et al. [41], if a sample size has sufficient information power depends on the aim of the study, sample specificity, use of established theory, quality of dialogue and analysis strategy. Our sample consisted of 10 women and two men, aged 27–66 years. The participants had different roles and specialisations: five were physiotherapists, two specialised in-patient physiotherapists, two were specialised community physiotherapists and three were leaders from a hospital, intermediate care and community health service. Their work experience ranged from 3 to 42 years. Although three were employed at an acute geriatric ward in a large Norwegian hospital, nine were employed as primary healthcare service providers in the community.

The current study was approved by the Regional Committee for Medical and Health Research Ethics (approval number 2015/1005 REC south-east D). The study followed the Declaration of Helsinki guidelines for research ethics when involving human participants, including informed consent, consequences and confidentiality [37]. The participants were informed about all aspects of the study, background, aim, and procedure, including information that the interview was recorded. The participants were informed that participation was voluntary and that they could withdraw at any time provided that the data related to them had not yet been analysed or published. All participants provided informed written consent, and none of them withdrew.

### Data collection

To answer the research question, individual semistructured interviews and a focus group interview as described by Creswell and Poth [42] were conducted. The last author collected the data from September 2016 to September 2017. In total, four individual semistructured in-depth interviews were conducted with physiotherapists who had participated in the OEP.

### Semistructured in-depth interviews

To explore physiotherapists’ opinions and personal experiences with the OEP, as well as their views about successful KT of the evidence-based fall programme—OEP—in the community, we conducted semistructured in-depth interviews. These interviews were conducted after the focus group interviews. The information generated from these interviews was intended to enhance our understanding and knowledge about the translation of research-based knowledge (e.g., the factors that facilitated or impeded the KT of the evidence-based OEP). Four clinical physiotherapists (women = 3, men = 1) who were employed in the community of Oslo/Akershus were interviewed. Except for one physiotherapist who also participated in the focus group interview, all participants were interviewed only once.

The last author began the semistructured in-depth interviews by posing general questions and eventually presented progressively more specific questions about their perceptions of OEP and their beliefs, confidence, and knowledge about the successful KT of the evidence-based OEP. We used a semistructured interview guide (see Additional file 1), supported by follow-up questions. Each interview lasted for 60 to 90 min and was facilitated by the last author. The physiotherapists who worked for the included municipalities were interviewed by the last author in their respective offices. All the interviews were audio taped.

### Focus group interview

A focus group design invites participants to communicate, interact and share their experiences with one another [43]. This method facilitates and captures spontaneous and informal discussions about participants’ experiences and perceptions because it provides participants the opportunity to add further information to others’ statements.

The focus group interview was conducted at the hospital, and nine physiotherapists participated. The physiotherapists discussed the factors that contributed to successful KT of the evidence-based OEP within their community and the impact of organisational factors such as leadership, culture and contextual and human resources. At the end of the focus group interview, the participants were given the opportunity to freely comment on topics they felt needed to be raised. We considered that this approach would offer insights into different perspectives about if and how they consider OEP to be relevant. This information could play an important role in the successful KT of the fall prevention programme. The focus group interview lasted for 90 min and was recorded. The focus group interview was facilitated by the last author—a physiotherapist and researcher in the field of fall prevention.

### The context of primary healthcare

The Norwegian healthcare system is predominantly state funded and divided into specialist and primary healthcare. Primary healthcare includes home-based services and nursing homes, whereas specialist healthcare includes state-owned hospitals, which are further divided into four regional health authorities [44]. The acute geriatric ward refers to a department that has an independent physical location and structure and that consists of a specialised multidisciplinary team that is directly responsible for the care of older adults with acute medical disorders [45]. All patients received home-based healthcare after discharge from the hospital. In the current study, home-based healthcare refers to the care that community-paid health workers provided to home-dwelling older adults, for example, short-term rehabilitation to long-term assistance with the activities of daily living and the advanced treatment of chronic and terminal illnesses [46].

### Data analyses

We conducted a thematic analysis, as developed by Braun and Clarke [47]. This is a method for identifying, analysing and reporting patterns within qualitative data. It contains six steps: (1) familiarisation with the data, (2) generating initial codes, (3) searching for themes, (4) reviewing the themes, (5) defining and naming the themes and (6) producing the report. Subsequently, we examined the ideas, conceptualisations and assumptions that underlay the expressed content [47].

Qualitative research creates a unique relationship between the participant and researcher. Rather than attempting to remove the role of the researcher altogether, as is the case in quantitative research, qualitative researchers attempt to interpret, understand and describe information in a reflexive process [48]. We used an independent professional transcriber to transfer the interviews from speech to text. Each author analysed the data independently to preserve variability, encourage reflexivity and establish credibility. The authors are health professionals in physiotherapy and nursing and are professionally trained educators. The first author (HW) was mainly responsible for the initial phase of data analysis, which was assisted and guided by the last author (AB). The codes were distributed to the rest of the research team (DL, BA and KSR), and further analysis and interpretation were performed jointly. To ensure trustworthiness, the categorisation was discussed within the research team until a consensus was reached. Furthermore, the methods and results (including quotations) were described in detail, according to the recommendations for consolidating criteria to report qualitative interviews (i.e., COREQ) [49]. Examples of the coding strategy are presented in Table 1.

**Table 1:** Examples of coding strategy

Quotation	Initial code	Main theme	Overarching theme
*Leadership is about influencing this context: these understandings, these ideas, norms, and what the desires should be.*	The leader’s tasks and their meanings	Multifactor leadership—the importance of reinforcement, knowledge, goals and attention	The empowering leader as an anchor for successful KT, representing physiotherapists’ and leaders’ views about the role of organisational structure, leadership, culture, financial resources and competence in research-based knowledge, as well as how to enhance the clinical staff’s expertise in using research-based knowledge.
*The organisational culture must be characterised by a positive attitude towards quality improvements and quality work, show a willingness to risk and have tolerance for different perceptions, uncertainties, and mistakes, and encourage critical testing.*	Organisational culture and willingness to take risks	Potential for change in professional roles, as shaped by culture, context and type of practice	
*I think the will is there; but little time and poor economy mean that there is a lack of time to acquire new research, time for additional education, as well as skills enhancement, time to read research, learn to do literature search and interpret the results, as well as (provide) administrative and financial support for implementing changes based on research results.*	Conflict between clinical practice and professional development	Knowledge translation—the tension between real-life capabilities, optimism and learning	
*In this field, one must also think about what is needed (for) behaviour change. Then it may be nice to take the starting point in behavioural change theories. What (does) it take to change behaviour? I don’t remember all the theories, but one of them is linked to (the) social cognitive theory of Bandura.*	Behaviour change	Different types of support—environmental resources and social influences of importance for regarding successful KT in fall prevention in the community	

In qualitative research, the researcher is an integrated part of the research process, shaping the collection of data and interpreting and explaining or describing human behaviour from the perspective of those participating in the study. Engaging in an intellectual process through which the knowledge and experience of those under study is juxtaposed with the knowledge, sensitising concepts and experience of the researcher, the findings of the research is clearly a joint construction; both parties work together to generate the research findings. Reflexivity demands an assessment of the influence of the researcher’s background, perceptions and interests on the research process [43]. It is important that the researcher is aware of his or her preunderstandings ahead of a research project. The term preconceptions implies the ideas, values, work experience, theoretical knowledge, life experience and motivation, along with the expected findings, the researcher brings into the project [50]. Preunderstanding is usually an important part of the researcher’s motivations for initiating a project on a specific topic. However, aspects such as age, gender and ethnicity might function as barriers or facilitators within research methods such as observation and interviews [51]. Thus, one’s preunderstanding can strengthen the project in the best-case scenario but may limit the researcher’s horizon and ability to learn from the data in the worst-case scenario [50]. The last author who conducted the interviews had a background as a physiotherapist and was familiar with the ‘language’ of the research context. However, this risk was acknowledged by the authors, and they purposefully maintained their awareness about how their preconceived notions may affect the study findings during both the interviews and data analyses.

## Results

The analysis resulted in four main themes: (1) multifactor leadership—the importance of reinforcement, knowledge, goals and attention; (2) potential for change in professional roles, as shaped by culture, context and type of practice; (3) knowledge translation—the tension between real-life capabilities, optimism and learning; and (4) different types of support—environmental resources and social influences of importance for regarding successful KT in fall prevention in the community. These main themes formed the overarching theme: the empowering leader as an anchor for successful KT, representing physiotherapists’ and leaders’ views of the role of organisational structure, leadership, culture, financial resources and competence in research-based knowledge, as well as how to enhance the clinical staff’s expertise in using research-based knowledge. Our findings suggest that the need for anchoring and support in contextual factors, organisational structure and culture is crucial for successful KT.

### Multifactor leadership—the importance of reinforcement, knowledge, goals and attention

The informants noted the importance of supportive and perseverant leadership in the never-ending process of KT in fall prevention (i.e., the OEP). They stated that in general, success in KT depends on a proactive leadership style focused on having sufficient knowledge about the intervention (i.e., the OEP).

The leaders’ dialogue was an issue discussed during the interviews. The participants emphasised that the leader’s voice and communication should reflect motivation and a ‘drive’ for KT of the OEP. As the practitioner’s head, messenger and advocate in the process, the leader’s speech must be filled with visions and motivation. The leader’s position and physiotherapists’ desires legitimise the action of power through knowledge and communication. One of the focus group participants stated the following:

*The leader must show interest and create glow, not resignation.*

The participants described the leader as a facilitator for the KT process of the OEP. Leadership does not take place in a vacuum; rather, it is an interaction with the practitioners and the context. This is also the foundation of the barriers and facilitators of the service. It is the leader’s job to affect the context and systematically shape the alteration process according to these factors. One participant in the focus group stated the following:

*Leadership is about influencing this context: these understandings, these ideas, norms and what the desires should be.*

According to the physiotherapists, one possible solution was a strong partnership between researchers, health personnel and others who work with quality development because doing this might increase insights into and understanding of the field. The participants emphasised the importance of capacity and competence at all levels, from leaders to health personnel. The informants also said that leaders must be good ‘sellers’ to sell the changes and create anchoring and involvement in the OEP. Their job is to engage the staff to communicate their KT strategy of the OEP. The properties can be compared with the mandate of stakeholders and opinion leaders. The leader should communicate and persuade the employees about their opinions and goals. The physiotherapists also expressed a need for closer cooperation with the leaders and a desire for participation in quality development. One physiotherapist said the following:

*Leadership should prioritise to a greater extent that practitioners can participate and actively participate in cultivating a culture for research-based knowledge and the development of practice*.

The service must be sustainable and have sufficient capacity and resources to carry out the KT process of the OEP. One crucial factor is that the context should be adapted and support this change. The participants stated that it is the leader’s responsibility to organise the leadership of the operation during the KT process of the OEP. Another important factor for success is the leader’s powerful position. Knowledge gives the leader authority. According to one of the physiotherapists, the action of power through communication and dialogue is essential for the OEP to succeed. As an example, one participant highlighted the leader’s demands for the employees to provide EBP and OEP. Both employees’ lack of interest in research and their interest in best practices is the leader’s responsibility. Thereby, the leader’s mandate is to motivate and keep the ‘zeal’ up and motivate the employees regarding the creation of an EBP for the OEP. The success of the OEP cannot be achieved without active leader support. Therefore, the leader must have an understanding and knowledge of what is to be implemented and understand the extent of it. One physiotherapist described it as follows:

*It requires a lot of knowledge and interest to keep updated. It is also a managerial responsibility that the employees are interested [OEP] in and that they actually follow and deliver a practice that is based on updated knowledge [such as OEP]. It is demanding, almost impossible.*

The leader’s role and responsibilities are diverse and are applied within a context. However, the leader’s role often ends someplace ‘in-between’ during the KT process of the OEP. Collaborators can control what leaders should spend their time on, while internal resource shortages can reduce leaders’ priorities. One focus group participant said the following:

*Partners, competitors, and legislators may seem to govern what leaders should spend their time on and what they should be concerned with, while internal resource deficiency can reduce managers’ priorities to questions.*

Quality improvement encompasses healthcare services at all levels. A participant described the importance of several factors, such as partnership, capacity and competence, in the different levels of the health service to improve the quality of the services. One key component is that all three factors involve human interaction. Thus, the dialogue becomes an important tool during the KT process of the OEP. One participant stated that it is about a common understanding of the purpose of the KT of the OEP because it may enhance the quality of the services. One focus group participant described it as follows:

*This is also closely linked to competent management, collective competence, and a collaborative climate.*

The informants agreed that KT of the OEP is a comprehensive process. To have a well-planned strategy for how to implement the OEP in clinical practice was proposed as important regarding successful KT. In addition to sufficient resources and necessary preconditions, there is also a need for a certain degree of standardisation and experience of control over the process. One focus group participant stated the following:

*I think the importance of programme integrity is related to the evidence- based intervention [OEP] and depends on securing necessary resources and preconditions as well as establishing appropriate support or supporting systems for new or changed practices focusing on the wanted future. This depends on the extent to which new practices [OEP] are supported by leadership, and whether there is sufficient motivation and support among the staff to get started … awareness of this is important.*

Implementing a new programme such as the OEP in clinical practice implies an alteration process, which requires maintenance and continuous work by the organisation*.* One focus group participant described that translation of the OEP is about making the system collectively sustainable by making the changes at different levels of the service system:

*Implementing is a continuous process where the goal is to establish and maintain practice based on research, clinical expertise, and patient values. It may involve both specific practical activities to more holistic processes, allowing individuals, teams, and organisations to change. It involves increased awareness about the need for changes, the importance of leadership and project management, how relationships are built, communication, and the importance of the local context and ongoing monitoring and evaluation.*

The participants highlighted the importance of having a vision for KT of the OEP because this would be important in the future alteration process. This requires management at a high level. Commitment by the organisation as a unit clarifies the direction of the process and how it is to proceed. The KT process is a never-ending process. To have a vision will also motivate health practitioners because it shows a way forward and may prevent endless and unnecessary discussions among the employees in the organisation. However, challenges may still arise. Furthermore, the participants highlighted that every single employee in the organisation needs to have a desire to participate. One participant in the focus group interview noted the following:

*There must be a result out of the commitment—something concrete—not just talk. It must be shown from the leader that there is a way forward. We see that the steps we take lead to goals … those who will participate in this practice should be ready for it [OEP]—a plan must be made.*

The participants stated that the KT process of the OEP requires that the right people be set to drive the process. It could be internal employees such as leaders or stakeholders; however, external consultants were also proposed. A key point is that they must have knowledge and competence of what is to be implemented, as well as about the KT process. Knowledge gives leaders credibility, which is important when they are in a dialogue with employees. Further, the participants highlighted that leaders’ ability to combine information with social influence and direct contact with the practitioners of the interventions is a crucial feature and qualification. One focus group participant expressed the following:

*Of course, it is an advantage if the leaders have practical experience with the intervention they provide. It creates trust. They must have legitimacy, be credible, and have good communication skills that are appropriate to the intervention as well as the receivers.*

Those who are responsible for KT of the OEP should engage the entire organisation and include everyone on the team and ensure that everyone is informed and involved in the process. There must be holistic ownership of the process. This requires good change management and that the change is implemented smoothly. The participants highlighted that the leader’s knowledge about and compliance with the organisation’s structure, values and goals are vital.

### Potential for change in professional role shaped by culture, context and type of practice

All informants suggested that the professional culture has various manifestations, such as stories, routines, formal and informal practices, jargon and physical arrangements. This indicates that the culture is part of the framework used to make sense of their everyday interactions of the importance to KT of the OEP. The influence of professional culture on the success of KT revealed a focus on employee autonomy and professional outcomes.

The cultural factors described in the interviews were the importance of innovation, support, social responsibility, competitiveness, stability, performance orientation and reward orientation. The physiotherapists described the health service as a social collective in which knowledge is developed and negotiated. Norms, values and visions are developed through social interaction and can both facilitate and inhibit KT of the OEP. The use of research and academic discussions can motivate professional development. The participants noted that internal conditions such as culture are crucial for how an organisation integrates new knowledge and the OEP:

*To change practice depends on the openness and culture of evidence-based practice.*

A physiotherapist said the following:

*Perhaps learning with and from each other, one can actually change perspective.*

One physiotherapist from the semistructured in-depth interviews described the KT process of the OEP as follows:

*Knowledge translation [of OEP] is about learning—both as an individual and (as a) more collective process or group process.*

The created culture in the workplace forms the basis for curiosity and motivation for further development. The ability to be critical and reflect on one’s own practice is noted as an important factor in the work culture. The service is drawn between the requirements for professional updating and everyday routines and priorities. This can delay the development and innovation of the service. Therefore, it is important that the changes that to be made are understood and experienced as relevant to the development of the service. The research must be linked to the daily needs of the practice and support everyday work. In addition, the change must also be feasible according to the resources and capacity of the service. Tailored interventions seemed to be of importance for KT of the OEP. One focus group participant stated the following:

*It must be noted—there is a need for knowledge, which must be adapted to the current context barriers and facilitators for the use of knowledge [OEP]. Mapping the process must be attempted so one can possibly choose and customise an implementation intervention that can be put into use and then evaluated and hopefully lead to a sustainable practice that all users think is okay.*

KT of the OEP is about change processes that are influenced and ordered by many different actors and emerged from various factors at different levels in the organisation or service. To achieve a successful change it is important that everyone in the service has an inherent commitment. One must have an interest and inner motivation to use research such as the OEP. Additionally, the service—as a collective—must be receptive to changes. A consensus should be the basis for all decisions and should be governed by the understanding and acceptance of ambiguity. One physiotherapist stated that one must look beyond one’s own attitudes and views and be open to new perspectives:

*Effective communication is not about literal transfers of meaning. Trying to remove any ambiguity is useless. It can be fruitful to try to find a framework that helps (reduce) the ambiguity to inspire exploration and curiosity. We must accept that we understand things differently and that somebody is sometimes perceived not to fully understand what it is about. Ambiguity and acceptance of different degrees of understanding provide the opportunity for negotiation. Practice is not about common beliefs understood as the same mental size. Unity, in common sense—in the literal sense—is not a prerequisite for mutually engaged in practice or their results. We can never control that. It must be brokered in terms of implementation.*

Another physiotherapist suggested that the KT process of the OEP requires mediation between several partners. It is about creating relationships and cooperation between practice, researchers and the environment based on common features and characteristics. Furthermore, the participants expressed the view that if there was any resistance to change, this could be because of a climate that is not generally change-oriented or that the organisational structure regarding the ability of the service to undergo change is not sustainable. The changes must be consistent with how the framework of the organisational structure. One physiotherapist during the focus group interview said the following:

*The relationship between clinical practice and research-based knowledge [OEP] is an important consideration. I think it’s important that it [OEP] doesn’t require too much change in clinical practice. If the employees resign their jobs, others need to take over. It requires organisation, as well as a willingness to constantly updating.*

Strategies that can translate the OEP into action and lead to programme integrity are essential. Strategic plans and policies will ensure the quality of the process and take into account unforeseen events, as well as other challenges that the individual service faces. As one participant asserted, ‘Better to be safe than sorry’. This will prevent instability and conflict, which can be a barrier to successful translation of the OEP. The individual’s loyalty to the work tasks, as well as ownership where the focus must extend beyond the core tasks, is described as a determinant in the translation process. One focus group participant said the following:

*The translation programme must meet a current need in a way that is consistent with the organisation’s core values. Obtaining the value base is not always easy; but there must be a broad consensus in the staff group on the need for change of practice, support for implementing a specific programme of measures, and the willingness to commit efforts over time, in other words, is central to success.*

Reflectivity over one’s competence and its effect also seem vital. The participants referred to themselves as maniacs who sometimes experience changes as threatening. One focus group physiotherapist said the following:

*We are some madmen and we have to take care of everyday life’s nailed, or more or less nailed, routines. The unknown can be perceived, consciously or unconsciously, as scary or threatening.*

Furthermore, all the participants agreed that the service must also be positive to changes and endure uncertainty and errors. As one focus group participant expressed the following:

*The organisational culture must be characterised by a positive attitude towards quality improvements and quality work and show a willingness to risk and have tolerance for different perceptions, uncertainties, and mistakes and encourage critical testing.*

### Knowledge translation of research-based knowledge—the tension between real-life capabilities, optimism and new learning

Acceptance of research-based knowledge and the OEP as related to the everyday reality and optimism about the new practice that a person can put to constructive use is important for successful KT. In addition, confidence that the desired goal is clear and achievable was also revealed as important in both focus group and the semistructured in-depth interviews. The participants mentioned that feeling connected and empowered is a matter of significance, as well as the capabilities in the form of properties that can stimulate attention to and then use knowledge. Communication based on shared decision making and consideration of the different external context factors affect the quality of KT.

The understanding of quality improvement seemed to be of importance for the KT process of the OEP; nevertheless, in general, the physiotherapists found that knowledge about EBP and research use was given a lower priority. Usually, what KT requires seems to conflict with the practitioners’ daily activities. Service operation is based on procedures and rarely enough innovation. Several of the participants generally expressed a lack of time to cater to KT and its necessary competence development. One focus group participant acknowledged this by stating the following:

*I think that possible barriers regarding translation are primarily about lack of time and competing tasks or a lack of clear priorities between tasks and expertise. There is hardly time. Time pressure and other commitments can make it difficult to release resources to translation [of research in general].*

Tailored interventions, where the strategies are designed to address the service’s challenges, increase accuracy regarding KT. Several participants described the importance of mapping the strengths and weaknesses of the service, as well as uncovering barriers and facilitators in the process of KT of the OEP. One focus group participant highlighted the following:

*I see that a number of conditions facilitate or inhibit good translation [of OEP]; but it is unclear to me which factors are critical or most important. It depends where one is in the translation process and maybe different translation components can compensate for one another: if it fails at one point, this can be compensated by strengthening other components. Poor training quality can to some extent be offset by good guidance. The relationship between programme effect, translation quality and conditions that affect translation [of OEP] is an important focus.*

The participants explained that learning has several dimensions in the KT of research and the OEP. One must focus on what people know about research related to fall prevention in general, as well as the OEP, and what matters for learning and for the KT to work, as well as the context that provides a framework to understand and enable learning. Thus, limits and routines can negatively impact development. Despite this, there was motivation for quality improvement and professional updates. One focus group participant said the following:

*I think the will is there; but little time and poor economy mean that there is a lack of time to acquire new research, time for additional education, as well as skills enhancement, time to read research, learn to do literature search and interpret the results, as well as (provide) administrative and financial support for implementing changes based on research results.*

Some participants shared their scepticism about the investment in the general KT process and suggested that piloting could be a solution for uncovering the benefit for the organisation. One focus group participant described that external requirements, such as finances and demands for efficiency, can create a fear of trying to implement research-based knowledge, for example, with the OEP, because of unfortunate outcomes:

*We need to reduce the fear of failure. Making mistakes must not always be seen as a fiasco but considered in relation to intentions and concrete descriptions of what went wrong and why.*

If the field and service are to be developed, one must look at the research as a starting point to dare to take chances. One physiotherapist who participated in a semistructured in-depth interview said the following

*It’s about trying out. You have to start with the starting point. If not, the future of this discipline is dark.*

The participants also described how the policy affects current practice. The participants asserted that the politicians are governed by their own political interests and do not show interest in the development of theoretical and clinical knowledge. They expressed a desire for cooperation with politicians because politicians should gain insights into the practice field and the challenges practitioners currently face. However, the politicians’ point of view for change is also an important issue because grants are often crucial to research and KT. One focus group participant said the following:

*Our perspective of the translation and interest in research is influenced by challenges in the society that are placed on the public agenda by politicians, experts, and researchers, professions, and other stakeholders, as well as which solutions become relevant in different time periods. Professions should be able to document their results when it comes to financial incentives and assist the politicians with their expertise*.

Communication, partnerships and collaboration across the levels of the health services were also proposed as some of the many solutions for ensuring the quality of the services. The knowledge that emerges from the collaboration must be given to all healthcare employees, who can then promote the dissemination of the intervention. One focus group participant said the following:

*I think that partnership between healthcare professionals, leaders who are responsible for quality assurance, other decision makers, and translation researchers is needed. I believe that the translation research could be promoted and make important contributions to fall prevention as well as other evidence-based healthcare services.*

### Different types of support—environmental resources and social influences

For effective KT of the OEP, the connections between the sender and receiver are essential. The participants highlighted various forms of support, such as guidance, behavioural theories and leadership support, describing them as ‘tools’ that could assist in supporting and facilitating the KT process. The participants stated that any circumstances that encouraged their development of skills, abilities and adaptive behaviour were important. The interpersonal processes that can cause changes in their thoughts, feelings or reactive patterns—by which they attempt to deal with research-based knowledge—are critical.

A common feature of the ‘tools’ is that they all include knowledge and evidence. The participants highlighted both material and human support. Weaknesses or deficiencies in the support system will constitute a barrier to the various transitions in KT. The identification of barriers and facilitators was also highlighted as a tool and support during the KT process. The participants described guidelines as the link between research and practice, and they adapted to the intended target group. Furthermore, the need for knowledge about how these should be implemented was highlighted by the physiotherapists. One focus group participant suggested that the challenges regarding KT might involve behaviour change:

*In this field, one must also think about what is needed (for) behaviour change. Then it may be nice to take the starting point in behavioural change theories. What (does) it take to change behaviour? I don’t remember all the theories, but one of them is linked to (the) social cognitive theory of Bandura.*

The participants stated that to implement EBP in an organisation, the staff must have competence in the field. They described that the service must facilitate the raising of competence and facilitate the use of knowledge among practitioners, here highlighting various approaches such as e-learning, practice visits and courses. One focus group participant said the following:

*One way to get this knowledge in use is by teaching people—reminding them how to work evidence-based … maybe we also have to find a way to raise awareness of evidence-based practices and how to communicate—how to take the user’s and practitioner’s understanding as a starting point.*

During the interviews, several participants emphasised the importance of administrative or managerial support, programme managers’ expertise, training, guidance and practical technical assistance to sustain high-quality KT over time. Nevertheless, interventions tailored to combat local and individual barriers seemed vital for success. The physiotherapists highlighted and suggested theories and methods that will provide insight into the underlying factors that act as a barrier or facilitator for implementation and KT.. One focus group participant expressed the importance of identifying practitioners’ attitudes to EBP concerning whether the service is ready for change:

*It may also be important to get an idea of the attitudes to evidence-based practice and how familiar they [practitioners] are to the concept.*

Several of the participants highlighted the end user—the patient—in relation to the treatment credibility as a decisive factor for KT; they stated that if the user is not available, the sender of the information will be left without a receiver. During the focus group, it was also discussed whether the recruitment of the target group was achieved based on the objectives of politicians, leaders and other stakeholders. One focus group participant stated the following:

*No less important for the translation, those who need the intervention must be available and participate. It will probably save individuals from suffering and the communities for the costs related to fall. The price associated with falls, fractures, and hospital admissions is not small. Politicians have a sense of economy savings; but the problem often is different budgets and politicians do not see the context. Recruiting is difficult. Which health professionals should (be) recruit (ed) is a big question.*

## Discussion

To the best of our knowledge, this was the first to investigate physiotherapists’ and leaders’ views on translating the evidence-based fall prevention programme Otago into practice at the community level. The current study aimed to explore the views of physiotherapists in clinical practice and their leader’s views on the importance of organisational factors such as leadership and contextual and human resources regarding successful KT of the OEP in a Norwegian community. The overall results have highlighted the need for consensus within the healthcare organisation regarding KT of research-based knowledge, which also involves negotiation, practical considerations, readiness to change and leaders’ role as motivators and facilitators. In addition, the overall results highlighted that successful KT of the OEP is related to how the leader perceives the nature and type of evidence-based fall prevention programmes, the quality of the context in which evidence is introduced and the way the leader facilitates the KT process. We will further discuss our findings, here with an emphasis on leadership, context, culture and human resources. As stated in the discussion, these factors are strongly related to each other.

Our findings resonate with the conceptual framework of the integrated-Promoting Action on Research Implementation in Health Services (i-PARIHS), which aims to represent the dynamic interplay of the factors that influence successful implementation [52]. i-PARIHS proposes that the successful implementation of evidence into practice is a function of the evidence to be implemented, the context in which implementation takes place and facilitation of the implementation process. According to our findings, KT of the OEP is a comprehensive process that includes anchoring staff’s research-based knowledge in clinical practice. Facilitating the organisation’s framework factors and cultural conditions regarding KT of the OEP was also highlighted as crucial for the process. Competence among the staff, including the leader’s research-based knowledge about the OEP and how to manage the KT process, is essential. Readiness to change in both the organisation and among the clinicians is of great importance. Our findings demonstrate that the leader and context play an important role in understanding the successful use of the OEP. The leader must be conscious regarding the readiness of the healthcare organisation to implement research-based knowledge by examining certain contextual factors. It is a leader’s job to affect the context and systematically shape the alteration process according to certain barriers or facilitators. Leadership is about positively influencing the context: the understandings, the ideas, norms and what the desires should be. This implies that the leader must appear as a ‘seller’ of changes and create anchoring and involvement, as well as engaging the staff and communicating the KT strategy. According to the i-PARIHS framework, the active role of facilitation in addressing the attributes of context that can inhibit or promote implementation also suggests that there is an interaction between facilitation and context. Facilitators adapt what they do to fit local needs and conditions [52]. According to Squires et al. [29], the context is generally a neglected problem in implementation science. The context interacts, affects, modifies, simplifies, restricts or contributes to the success of the intervention [29]. In accordance with findings from previous research [53–55], our findings regarding the translation of the OEP into practice emphasised that time, resources and priorities were required. Additionally, the participants in our study emphasised the importance of capacity and resources while also highlighting knowledge and understanding of the components of quality improvement processes, as well as understanding what EBP and the OEP are about. Our findings highlighted that in general, the use of research-based knowledge was less prioritised in daily practice, and what KT requires, such as time to read research articles, seems to conflict with practitioners’ daily activities. The participants described that services are currently based on daily routines, and often, there is little time for innovation. Several participants expressed a lack of time to cater to KT and the necessary competence raising. The marginalisation of evidence in clinical practice regarding reading research articles, as demonstrated in our findings, is also supported by Melnyk et al. [56], who reported that top quality and safety in hospitals and health services were critical; yet EBP was ranked as a low priority. Morgan [57] has claimed that leaders who want to promote change need to create a culture in which attitudes stimulate openness, self-criticism, proactive initiative, renewal and so forth [57]. According to our participants, the created culture in the workplace forms the basis of curiosity and motivation for further development, as well as for the OEP. The participants expressed that the ability to be critical and reflect on one’s own practice is an important factor in the work culture. As such, regarding the implementation of the OEP in clinical practice, the high demands of the individual employee’s professional development and motivation for change is a requirement. Hence, to achieve the changes needed to implement the OEP in practice, it is vital that everyone working within the service has an inherent commitment. The physiotherapists in the current study suggested that one must have an interest and motivation to update their knowledge status. According to Senge [58] and in line with our findings, personal mastery implies that one has knowledge, can practice specific skills and engages in the continuous development of personal visions, goals and knowledge. Because research is constantly evolving, the capacity of the individual to acquire new knowledge and contextual factors (e.g., time), in general, will also be a decisive factor in KT of the OEP. The individual health worker cannot be forced into innovation/renewal; however, leaders can make important contributions here by creating an environment that incites personal growth. This implies creating an organisation where it feels safe for the individuals to raise questions about practice and established routines [59]. Furthermore, people with a high degree of personal mastery are more committed to continuous personal development [60]. For any person who advocates for personal development, it is critical to have support in the environment. By highlighting the importance of leadership, our findings resonate with the results of prior studies [27, 61], which have referred to factors such as leadership as being related to specific leader behaviours that support EBP use. Another study showed that the translation climate, which is described as perceptions on whether EBP use is expected, supported and rewarded, may also play an important role in successful EBP [62].

Leadership and organisational climate have been described as malleable organisational characteristics and may be more proximal to KT success than other organisational factors such as culture (shared norms and behavioural expectations that guide how work is prioritised and completed in the organisation) and staffs’ shared perceptions of the impact of the work environment on their personal well-being [63–65]. As demonstrated in previous studies [27, 61], our findings have shown that changes in practice require anchoring and leadership. The participants emphasised that research and EBP in fall prevention are constantly changing, meaning that the KT process is a continuous one. The current findings highlight that culture, including norms, values and visions, can both hinder and facilitate the effectiveness of an organisation. According to Schein and Schein [66], leadership is the fundamental process that shapes and transforms organisational culture. Further, Schein and Schein [66] describe how culture and leadership are two sides of the same story: leaders are both influenced by and influence the culture itself. Therefore, understanding how the culture affects the employees of an organisation and how the leader can influence the culture is important. However, as noted by Morgan [67], hierarchical organisations tend to fight, debate and prevent chances from being made [57]. According to our findings, a hierarchical organisation is an undesirable situation although our participants legitimatised the exercise of power through knowledge and communication. Although resistance can prevent or delay the desired change, Hennestad et al. [67] have stated that healthy scepticism can be regarded and used constructively in the change process. In the current study, the participants consistently expressed that ambiguity and new perspectives that compromise one’s own attitudes and views can contribute to innovation/rethinking and change.

Our findings have emphasised that the employees and organisation must obtain a consensus for all decisions adopted so that KT of the OEP can succeed. The importance of involving employees in these processes—to create a common understanding of the commitment to the relevant change—is also supported by Roland and Westergård [68]. Furthermore, a systematic review by Gifford et al. [31] highlights the importance of managerial engagement, collaboration and negotiation to promote the use of research among employees in clinical practice. Accordingly, our findings have shown that the organisation must dare to show a willingness to take risks, incorporate/hold uncertainty and tolerate mistakes, as well as critical testing innovative interventions. According to Morgan [57], employees taking part in a change process need to be supported, which entails the tolerance of mistakes. However, the KT literature in general acknowledges that KT is challenging, and it may be related to barriers in the organisation’s culture [59]. Li et al. [59] note that organisational culture was the most reported element affecting the translation of research-based knowledge. Regarding KT of the OEP, the participants in our study highlighted the importance of empowering leadership, including leading the KT process and motivating employees to work in a research-based way, promoting a culture where the norms and values for quality improvement are central, hence facilitating the clinical context to implement EBP. In line with our participants, Aadland [69] uses the expression ‘culture is in the walls’. According to Geertz and Darnton [70], the culture is the autopilot of the service. Therefore, culture can be inertial [71, 72] and affect change with resistance. Schein and Schein [66] emphasise the importance of the organisation being ‘softened’ and mindful of changes. Management to succeed with the change of culture is crucial to success [59].

In line with Cerderbom et al. [73], our findings suggest that organisational characteristics and human resources provide an important context for understanding the successful use of the OEP. Although our findings show that the different organisational characteristics may appear to act independently, the effects appear to be interrelated in relation to KT of the OEP. A supporting organisation that is sustainable, has sufficient capacity and resources and has clear leadership seems to have a satisfactory climate for change. Richardson and Vandenberg [74] state that positive leadership is associated with a climate of involvement, in which followers feel involved in problem solving and organisational decision making. Thus, leadership is a prerequisite and an antecedent of organisational culture and climate. Leadership is crucial for KT of the OEP as an organisational climate that supports EBP implementation and where sustainability can facilitate implementation [75].

A model of situational leadership has bene developed by Hersey and Blanchard [76], who define management as ‘a function of the leader, employees, and a serious situation-specific variable’*.* Situational leadership distinguishes primarily between two main types of leaders: authoritarian and democratic styles. Authoritarian leaders focus on task orientation, while democratic leaders are most concerned with the participants and their relationships. In line with Hersey and Blanchard [76] description of the democratic leadership style, our participants expressed a need for closer cooperation with leaders and a desire for quality improvement. Human resources are important for employee participation, and its importance in quality improvement has also been described by Schein and Schein [66]. Further, according to Fullan [77], understanding one’s own contributions is important for the translation process. In addition, our participants emphasised the importance of assessing the organisation’s capacity and human resources. In line with our findings, Meyers et al. [78] state that changes in an organisation must be in accordance with the needs, and the intervention must be adapted. Hersey and Blanchard [76] emphasise the importance of human resources, stating that people are different and work differently across diverse situations. This also demands diverse management styles from leaders. A leader’s role must be adapted to the individual employee and the current situation. This statement is supported by the findings from the current study: the participants indicated the need for the leader to embrace the employees’ varied forms of motivation, knowledge and expertise. In addition, the participants in the current study emphasised the leader’s qualities, such as being supportive, motivating and inspiring, as crucial for the KT process of the OEP. According to Reichenpfader et al. [28], leadership and leadership support were perceived as modifier for successful KT. In a systematic review conducted by Li et al. [59], leadership was commonly reported as influencing KT outcomes. Schreiner [79] emphasises the importance of an active and committed leader who communicates while also highlighting that quality is important.

Regarding the implementation of the OEP, our findings show that to bring the whole organisation together, the leader should actively and visibly enter the changing process and be conscious of how the context is governed to contribute to successful KT. A leader who is involved in the actual KT process is an important factor in the success of realising EBP and the OEP [27]. Additionally, supportive leadership skills, including articulating visions, clarifying priority goals, managing inhibitors in the KT process and so on, are necessary to institutionalise EBP in an organisation [27]. These findings are in accordance with our participants’ statements, specifically that the leader must have an inherent commitment to what is to be implemented and that communication should come with confidence and intention. A leader’s behaviour should reflect the motivation and a ‘drive’ for what is to be implemented. In addition, there must be a strategy to achieve the goal. These findings are in line with the findings from Balogun [80], who assert that a leader’s role is being a guide between the intended and unintended consequences.

### Strengths and limitations

There are several limitations to the current study. The findings represent the views of 12 physiotherapists, including leaders, in one area of Norway. Therefore, the transferability of the findings may be limited to similar contexts where fall prevention is used within a health care service with physiotherapists. Furthermore, transferability will also be limited to the public health system, which differs from private institutions and services. For qualitative research, it is argued that participants should reflect the diversity of our culture and conditions, including race. The lack of diversity among the research participants might impede the transferability of the study results. Regarding the present study, all participants were ethnic Norwegian, making this a potential limitation for the study’s transferability to an international context. Furthermore, semistructured in-depth interviews with leaders might have contributed several perspectives and nuances to the findings. To gain knowledge about the KT process of the OEP in practice, using observation as a method could have contributed additional information. Another limitation may be that because of a lack of resources, an observer or independent moderator who could write field notes or ask several questions based on observations was not used. Despite these limitations, our data and findings are unique. Although generalisation of qualitative findings is not a goal of qualitative research, qualitative researchers strive for the transferability of qualitative research findings. To address this, we provided rich and detailed descriptions of physiotherapists’ views. The current study’s authors have backgrounds in nursing and physiotherapy, which influenced the study design, data collection and data interpretation throughout the research process. Research has shown that other healthcare professionals, such as nurses and occupational therapists, can administer the OEP [81, 82]; thus, our results might be important for them, even though only physiotherapists were interviewed in our study. Future studies exploring the barriers and facilitators to continued exercise are also important during the intervention phase, enabling older people to continue with lifelong exercise to reduce mortality and falls [13, 83].

## Conclusion

The current study advances our understanding of the translation and sustainability of evidence-based fall prevention programmes for patients in the community. The findings suggest that empowering leadership is of great importance for KT of research-based knowledge in providing a supportive culture and contextual factors. This requires good leadership qualities. The leader has an important role as a motivator to contribute to a consensus concerning implementing EBP. To influence organisational perspectives regarding the use of research-based knowledge, a leader’s own competence is relevant for the KT process and, thus, contributes to bridging the perceived gap between research and practice. With a growing demand for fall prevention programmes, we believe that our insights around the implementation of the OEP will inform others who are considering or in the process of integrating the OEP into their practice.

## Supplementary Information

**Additional file 1.**

## Data Availability

The data and material can be obtained by contacting the corresponding author.

## Abbreviations

EBP: Evidence-based practice
KT: Knowledge translation
OEP: Otago exercise programme
RU: Research utilisation

## References

[1] World Health Organization (2018). Ageing and health. World Health Organization.

[2] Folkehelseinstituttet (2018). Population in Norway.

[3] Gillespie LD, Robertson MC, Gillespie WJ, Sherrington C, Gates S, Clemson LM, et al. Interventions for preventing falls in older people living in the community. Cochrane Database Syst Rev. 2012;(9):Cd007146. 10.1002/14651858.CD007146.pub3. 10.1002/14651858.CD007146.pub3 PMC8095069 22972103

[4] Russell K, Taing D, Roy J (2017). Measurement of fall prevention awareness and Behaviours among older adults at home. Can J Aging.

[5] Child S, Goodwin V, Garside R, Jones-Hughes T, Boddy K, Stein K (2012). Factors influencing the implementation of fall-prevention programmes: a systematic review and synthesis of qualitative studies. Implement Sci.

[6] Grant A, Mackenzie L, Clemson L (2015). How do general practitioners engage with allied health practitioners to prevent falls in older people? An exploratory qualitative study. Australas J Ageing.

[7] Vieira ER, Palmer RC, Chaves PHM (2016). Prevention of falls in older people living in the community. BMJ..

[8] Hopewell S, Adedire O, Copsey BJ, Sherrington C, Clemson LM, Close JCT, et al. Multifactorial and multiple component interventions for preventing falls in older people living in the community. Cochrane Database Syst Rev. 2016. 10.1002/14651858.CD012221. 10.1002/14651858.CD012221.pub2 PMC6513234 30035305

[9] Stubbs B, Brefknkinger M. Wa S, Dehat works to prevent falls in community-dwelling older adults? An umbrella review of meta-analyses of randomized controlled trials. Phys Ther. 2015. 10.2522/ptj.20140461. 10.2522/ptj.20140461 25655877

[10] Goodwin VA, Abbott RA, Whear R, Bethel A, Ukoumunne OC, Thompson-Coon J (2014). Multiple component interventions for preventing falls and fall-related injuries among older people: systematic review and meta-analysis. BMC Geriatr.

[11] Petridou ET, Manti EG, Ntinapogias AG, Negri E, Szczerbinska K (2009). What works better for community-dwelling older people at risk to fall?: a meta-analysis of multifactorial versus physical exercise-alone interventions. J Aging Health.

[12] Sherrington C, Michaleff ZA, Fairhall N, Paul SS, Tiedemann A, Whitney J (2017). Exercise to prevent falls in older adults: an updated systematic review and meta-analysis. Br J Sports Med.

[13] Thomas S, Mackintosh S, Halbert J (2010). Does the ‘Otago exercise programme’ reduce mortality and falls in older adults?: a systematic review and meta-analysis. Age Ageing.

[14] El-Khoury F, Cassou B, Charles MA, Dargent-Molina P (2013). The effect of fall prevention exercise programmes on fall induced injuries in community dwelling older adults: systematic review and meta-analysis of randomised controlled trials. BMJ..

[15] Van Rhyn B, Barwick A (2018). Health practitioners’ perceptions of falls and fall prevention in older people: a Metasynthesis. Qual Health Res.

[16] Jones TS, Ghosh TS, Horn K, Smith J, Vogt RL (2011). Primary care physicians perceptions and practices regarding fall prevention in adult's 65 years and over. Accid Anal Prev.

[17] Shubert TE, Smith ML, Prizer LP, Ory MG (2014). Complexities of fall prevention in clinical settings: a commentary. Gerontologist..

[18] Carpenter CR, Lo AX (2015). Falling behind? Understanding implementation science in future emergency department management strategies for geriatric fall prevention. Acad Emerg Med.

[19] Li F, Eckstrom E, Harmer P, Fitzgerald K, Voit J, Cameron KA (2016). Exercise and fall prevention: narrowing the research-to-practice gap and enhancing integration of clinical and community practice. J Am Geriatr Soc.

[20] Guse CE, Peterson DJ, Christiansen AL, Mahoney J, Laud P, Layde PM (2015). Translating a fall prevention intervention into practice: a randomized community trial. Am J Public Health.

[21] Radecki B, Reynolds S, Kara A (2018). Inpatient fall prevention from the patient's perspective: a qualitative study. Appl Nurs Res.

[22] Sackett DL, Rosenberg WMC, Gray JAM, Haynes RB, Richardson WS (1996). Evidence based medicine: what it is and what it isn’t. BMJ..

[23] Bussieres AE, Al Zoubi F, Stuber K, French SD, Boruff J, Corrigan J (2016). Evidence-based practice, research utilization, and knowledge translation in chiropractic: a scoping review. BMC Complement Altern Med.

[24] Andrews D, Fong G, Hackam D, Li LJL, M M (2020). Guide to knowledge translation planning at CIHR: Integrated and end-of-grant approaches.

[25] Grimshaw JM, Eccles MP, Lavis JN, Hill SJ, Squires JE (2012). Knowledge translation of research findings. Implement Sci.

[26] Cullen L, Adams SL (2012). Planning for implementation of evidence-based practice. JONA..

[27] Stetler CB, Ritchie JA, Rycroft-Malone J, Charns MP (2014). Leadership for evidence-based practice: strategic and functional behaviors for institutionalizing EBP. Worldviews Evid-Based Nurs.

[28] Reichenpfader U, Carlfjord S, Nilsen P (2015). Leadership in evidence-based practice: a systematic review. Leadersh Health Serv (Bradf Engl).

[29] Squires JE, Aloisio LD, Grimshaw JM, Bashir K, Dorrance K, Coughlin M (2019). Attributes of context relevant to healthcare professionals’ use of research evidence in clinical practice: a multi-study analysis. Implement Sci.

[30] Melnyk B, Fineout-Overholt E, Gallagher-Ford L, Kaplan L (2012). The state of evidence-based practice in US nurses: critical implications for nurse leaders and educators. J Nurs Adm.

[31] Gifford WA, Squires JE, Angus DE, Ashley LA, Brosseau L, Craik JM (2018). Managerial leadership for research use in nursing and allied health care professions: a systematic review. Implement Sci.

[32] Jun J, Kovner CT, Stimpfel AW (2016). Barriers and facilitators of nurses’ use of clinical practice guidelines: an integrative review. Int J Nurs Stud.

[33] Melnyk BM, Fineout-Overholt E (2019). Evidence-based practice in nursing & healthcare : a guide to best practice.

[34] Worum H, Lillekroken D, Ahlsen B, Roaldsen KS, Bergland A (2019). Bridging the gap between research-based knowledge and clinical practice: a qualitative examination of patients and physiotherapists’ views on the Otago exercise Programme. BMC Geriatr.

[35] Lockwood C, Stannard D, Jordan Z, Porritt K (2020). The Joanna Briggs institute clinical fellowship program: a gateway opportunity for evidence-based quality improvement and organizational culture change. Int J Evid Based Healthc.

[36] Albrecht L, Archibald M, Arseneau D, Scott SD (2013). Development of a checklist to assess the quality of reporting of knowledge translation interventions using the workgroup for intervention development and evaluation research (WIDER) recommendations. Implement Sci.

[37] World Medical Association (2013). Declaration of Helsinki, ethical principles for medical research involving human subjects.

[38] Willig C (2008). Introducing qualitative research in psychology : adventures in theory and method.

[39] Rudestam KE, Newton RR. Surviving Your Dissertation: A Comprehensive Guide to Content and Process. London: SAGE Publications; 2007.

[40] Neubauer BE, Witkop CT, Varpio L (2019). How phenomenology can help us learn from the experiences of others. Perspect Med Educ.

[41] Malterud K, Siersma VD, Guassora AD (2016). Sample size in qualitative interview studies: guided by information power. Qual Health Resg.

[42] Creswell JW, Poth CN (2018). Qualitative inquiry & research design: choosing among five approaches.

[43] Jensen GM (2001). Using Qualitative Research: A Practical Introduction for Occupational and Physical Therapists. Physiother Theory Pract.

[44] Ringard Å, Sagan A, Saunes IS, Lindahl AK. Health system review, vol. 15. Norway; 2014. p. 162. [cited 2019 03.03] Available from: https://www.fhi.no/en/publ/2014/norway%2D%2Dhealth-system-review2/.

[45] Baztán JJ, Suárez-García FM, López-Arrieta J, Rodríguez-Mañas L, Rodríguez-Artalejo F (2009). Effectiveness of acute geriatric units on functional decline, living at home, and case fatality among older patients admitted to Hospital for Acute Medical Disorders: meta-analysis. BMJ..

[46] Beard JR, Officer A, De Carvalho IA, Sadana R, Pot AM, Michel JP (2016). The world report on ageing and health: a policy framework for healthy ageing. Lancet..

[47] Braun V, Clarke V (2006). Using thematic analysis in psychology. Qual Res Psychol.

[48] Wilding C, Whiteford G (2005). Phenomenological research: an exploration of conceptual, theoretical, and practical issues. OTJR..

[49] Tong A, Sainsbury P, Craig J (2007). Consolidated criteria for reporting qualitative research (COREQ): a 32-item checklist for interviews and focus groups. Int J Qual Health Care.

[50] Alvesson M, Sköldberg K (2009). Reflexive Methodology: New Vistas for Qualitative Research.

[51] Hammersley M, Atkinson P (2019). Ethnography: Principles in Practice.

[52] Harvey G, Kitson A (2016). PARIHS revisited: from heuristic to integrated framework for the successful implementation of knowledge into practice. Implement Sci.

[53] Ammouri AA, Raddaha AA, Dsouza P, Geethakrishnan R, Noronha JA, Obeidat AA (2014). Evidence-based practice: knowledge, attitudes, practice and perceived barriers among nurses in Oman. Sultan Qaboos Univ Med J.

[54] Sadeghi-Bazargani H, Sadegh Tabrizi J, Azami-Aghdash S. Barriers to evidence-based medicine: a systematic review. J Eval Clin Pract. 2014. 10.1111/jep.12222. 10.1111/jep.12222 25130323

[55] McConville A, Hooven K. Factors influencing the implementation of falls prevention practice in primary care. J Am Assoc Nurse Pract. 2020. 10.1097/jxx.0000000000000360. 10.1097/JXX.0000000000000360 32251034

[56] Melnyk BM, Gallagher-Ford L, Thomas BK, Troseth M, Wyngarden K, Szalacha L (2016). A study of chief nurse executives indicates low prioritization of evidence-based practice and shortcomings in hospital performance metrics across the United States. Worldviews Evid-Based Nurs.

[57] Morgan G (1988). Riding the Waves of Change: Developing managerial competencies for a turbulent world. Organ Stud.

[58] Senge PM (2006). The fifth discipline : the art and practice of the learning organization.

[59] Li SA, Jeffs L, Barwick M, Stevens B (2018). Organizational contextual features that influence the implementation of evidence-based practices across healthcare settings: a systematic integrative review. Syst Rev.

[60] García-Morales V, Llorens Montes F, Verdu A (2007). Influence of personal mastery on organizational performance through organizational learning and innovation in large firms and SMEs. Technovation..

[61] Harvey G, Gifford W, Cummings G, Kelly J, Kislov R, Kitson A (2019). Mobilising evidence to improve nursing practice: a qualitative study of leadership roles and processes in four countries. Int J Nurs Stud.

[62] Weiner BJ, Belden CM, Bergmire DM, Johnston M (2011). The meaning and measurement of implementation climate. Implement Sci.

[63] Aarons G, Ehrhart M, Torres E, Finn KN, Beidas R. The Humble Leader: Association of Discrepancies in Leader and Follower Ratings of Implementation Leadership With Organizational Climate in Mental Health. Psychiatr Serv. 2017:115–22. 10.1176/appi.ps.201600062. 10.1176/appi.ps.201600062 PMC5462527 27691380

[64] Aarons GA, Ehrhart MG, Moullin JC, Torres EM, Green AE (2017). Testing the leadership and organizational change for implementation (LOCI) intervention in substance abuse treatment: a cluster randomized trial study protocol. Implement Sci.

[65] Aarons GA, Ehrhart MG, Farahnak LR, Hurlburt MS (2015). Leadership and organizational change for implementation (LOCI): a randomized mixed method pilot study of a leadership and organization development intervention for evidence-based practice implementation. Implement Sci.

[66] Schein EH, Schein P (2016). Organizational culture and leadership.

[67] Hennestad BW, Revang Ø, Strønen FH (2012). Endringsledelse og ledelsesendring/ Change management and management of change. 2. utg. ed.

[68] Roland P, Westergård E (2015). Implementering : å omsette teorier, aktiviteter og strukturer i praksis/ implementation: to translate theories, activities and structures into practice.

[69] Aadland E (1994). Kultur i helse-, sosial- og utdanningsorganisasjonar/ culture in health, social and educational organizations.

[70] Geertz C, Darnton R (2017). The interpretation of cultures: selected essays.

[71] Heracleous L (2001). An ethnographic study of culture in the context of organizational change. J Appl Behav.

[72] Elsmore P (2001). Organisational culture: Organisational change? Gower.

[73] Cerderbom S, Bjerk M, Bergland A (2020). The tensions between micro-, meso- and macro-levels: physiotherapists’ views of their role towards fall prevention in the community – a qualitative study. BMC Health Serv Res.

[74] Richardson H, Vandenberg R (2005). Integrating managerial perceptions and transformational leadership into a work-unit model of employee involvement. J Organ Behav.

[75] Aarons GA, Ehrhart MG, Farahnak LR, Sklar M (2014). Aligning leadership across systems and organizations to develop a strategic climate for evidence-based practice implementation. Annu Rev Public Health.

[76] Hersey P, Blanchard KH (1969). Life cycle theory of leadership. Train Dev J.

[77] Fullan M (2007). The new meaning of educational change.

[78] Meyers D, Durlak J, Wandersman A (2012). The quality implementation framework: a synthesis of critical steps in the implementation process. Am J Community Psychol.

[79] Schreiner A (2004). Kom i gang. Kvalitetsforbedring i praksis/ get started. Quality improvement in practice.

[80] Balogun J (2006). Managing change: steering a course between intended strategies and unanticipated outcomes. Long Range Plan.

[81] Robertson C, Devlin N, Gardner M, Campbell A (2001). Effectiveness and economic evaluation of a nurse delivered home exercise programme to prevent falls. 1: Randomised controlled trial. BMJ (Clinical research ed).

[82] Lombard K, Desmond L, Phelan C, Brangan J (2019). Irish occupational therapists use of evidenced-based falls prevention programmes. IJOT..

[83] Finnegan S, Bruce J, Seers K (2019). What enables older people to continue with their falls prevention exercises? A qualitative systematic review. BMJ Open.

