# Supplementary material for: Otago exercise programme—from evidence to practice: a qualitative study of physiotherapists’ perceptions of the importance of organisational factors of leadership, context and culture for knowledge translation in Norway
Source: BMC Health Serv Res. 2020 Oct 27;20:985. doi: 10.1186/s12913-020-05853-8 (PMC7590709; doi:10.1186/s12913-020-05853-8)
Supplement: Supplementary file 1 — Additional file 1. [file 12913_2020_5853_MOESM1_ESM.docx]

**Sample questions that were posed to participants during the in-depth interviews**

| **Physiotherapists** | **Interview: 1 week after the completion of the Otago Exercise Programme**   - Can you please describe your experience participating in the evidence-based Otago Exercise Programme? - Can you please describe your views about the research-based knowledge that pertains to fall prevention? - What are the factors that contribute to the successful implementation of a research-based program such as the Otago Exercise Programme? - What do you think about the application of research-based knowledge in fall prevention interventions? - How can you and your college contribute to the application of research-based knowledge in fall prevention? - How can the application of research-based knowledge be improved? |
| --- | --- |
